# Eine sportliche Herausforderung

**DOI:** 10.1007/s43594-020-00024-3

**Published:** 2020-12-04

**Authors:** Michael Leyendecker, Nils Neuber, Peter Lautenbach

**Affiliations:** 1 Deutsche Sportjugend, Köln, Deutschland; 2 Westfälische Wilhelms-Universität Münster, Münster, Deutschland; 3 Deutsche Sportjugend, Frankfurt am Main, Deutschland

Liebe Leserinnen und Leser,

„Im Lockdown haben sich Kinder mehr bewegt als sonst“, titelte *Der Spiegel* Ende August und zitierte aus einer Studie, nach der Heranwachsende in der Pandemie andere Wege fanden, um Sport zu treiben, nachdem Schulsport und Vereinstraining ausfielen. Kann das möglich sein? Brauchen wir keine pädagogisch ausgerichteten Sportangebote mehr? Mitnichten! Die Studie zeigt lediglich, dass sich Kinder und Jugendliche gerne bewegen – und in der Not eigene Wege finden, um das zu realisieren. Die Studie fragt nicht nach Interaktion, Teilhabe oder Mitgestaltung im Sport. Sie fragt nicht nach der Qualität von Bewegungsangeboten für junge Menschen. Insofern ist ein pädagogisch verantworteter Kinder- und Jugendsport wichtiger denn je, nicht zuletzt weil bestehende Herausforderungen in der Coronakrise deutlicher hervortreten als zuvor.

So formulierte *Deutschlandfunk Kultur* im Juli: „Die Coronakrise wirkt wie ein Brennglas für gesellschaftliche Missstände – vielleicht öffnet sie aber auch den Horizont für mögliche Veränderungen.“ Tatsächlich werden soziale Ungleichheiten in der Pandemiezeit besonders deutlich. Kinder, die schon vorher wenig Unterstützung fanden, werden momentan noch weiter abgehängt. Corona verdichtet vorhandene Krisen. Was für die Demokratie, die Wirtschaft oder das Gesundheitswesen gilt, trifft auch auf die Kinder- und Jugendarbeit im Sport zu: Tiefliegende Probleme tauchen an die Oberfläche. Die Covid-19-Pandemie ist daher nicht nur für die Virologen eine Herausforderung, sondern auch für den Kinder- und Jugendsport.

In der zweiten Ausgabe von *Forum Kinder- und Jugendsport – Zeitschrift für Forschung, Transfer und Praxisdialog *greifen wir daher das Thema der Covid-19-Pandemie auf. Die Forschung zu ihren Auswirkungen auf den Kinder- und Jugendsport läuft, und bereits in diesem Heft erläutern neun Wissenschaftler*innen den Einfluss des Virus auf die Kinder- und Jugendarbeit im Sport und die daraus entstehenden Herausforderungen.

*Forum Kinder- und Jugendsport *unterstreicht in ganz unterschiedlichen Beiträgen, wie bedeutsam Bewegung und Interaktion für Kinder und Jugendliche sind und stößt den Dialog zwischen Forschung und Praxis an. Die vorliegende zweite Ausgabe enthält wieder drei Forschungsbeiträge. Zwei behandeln das Sportangebot in Ganztagsschulen. *Heike Gumz* gibt Einblicke in das Raum- und Bewegungserleben aus der Sicht von Kindern. *Sebastian Braun* und *Katrin Albert* befassen sich mit der Rolle von Sportagent*innen als Koordinationsinstanz zwischen Schule und Sportverein. Entsprechend unserem dialogischen Ansatz skizziert *Karl Schilling*, stellvertretender Vorsitzender im Turn-Klubb zu Hannover, in einer Projektskizze, wie der Verein vor Ort die Entwicklung von Ganztagsgrundschulen prägt.

Im dritten Forschungsbeitrag thematisieren *Kathrin Kohake* und *Alfred Richartz* die motivationale Bedeutung des Kompetenzerlebens im Schul‑, Breiten- und Leistungssport von Kindern. Ihre Untersuchung wird von gleich drei Fachbeiträgen umrahmt: *Tim Brentjes* schildert die erfolgreiche Trainer*innenausbildung im Kinderbasketball. Zur Kompetenzentwicklung im Skiverband hat *Bettina Haueisen* geschrieben. *Katharina Morlang*, dsj-Referentin für Bildung und Qualifizierung, ist Autorin des Beitrags zum Selbstverständnis von Trainer*innen in puncto pädagogischer Trainingsqualität. Er basiert auf Befragungen der noch unveröffentlichten Evaluation zum DOSB-Projekt „TrainerInSportdeutschland“.

Zurück zu Corona und zur Wissenschaft: *Gunda Voigts* schreibt über die Bedeutung von Freiräumen für Jugendliche in dieser Zeit. Aus dem dsj-Forschungsverbund antworten neun Wissenschaftler*innen prägnant aus ihrer jeweiligen Forschungsperspektive auf Fragen der Redaktion zu den Herausforderungen der Pandemie: *Ulrike Burrmann* (Integration), *Ahmet Derecik* (Partizipation), *Petra Gieß-Stüber* (Interkulturelles Lernen), *Detlef Kuhlmann* (Qualifikation), *Nils Neuber* (Informelles Lernen), *Alfred Richartz* (Pädagogik im Kinderleistungssport), *Bettina Rulofs *(Prävention sexualisierter Gewalt), *Jessica Süßenbach* (Kommunale Kinder- und Jugendsportentwicklung) und *Ralf Sygusch* (Persönlichkeits- und Teamentwicklung).

Die Engagementförderung ist schließlich Thema eines Beitrags, in dem die Vorbildfunktion des organisierten Sports für eine Juniorteam-Gründung außerhalb des Sports aufgegriffen wird. Geschrieben hat ihn *Rebecca Jung*, Mitglied im Juniorteam der Kinderhilfe Organtransplantation. Zu guter Letzt, aber in der Druckausgabe gleich zu Beginn platziert: In der Rubrik „Das aktuelle Stichwort“ gehen *Torsten Weber* und *Stefan Wagner* auf den Status der Nachhaltigkeitsbewegung im deutschen Sport ein. Nachhaltigkeit – auch eine Aufgabe, die durch Corona beeinflusst wird und einer authentischen Darstellung und umfassenden Berücksichtigung im Kinder- und Jugendsport bedarf.

Wir hoffen, auch mit der zweiten Ausgabe von *Forum Kinder- und Jugendsport* ein breites Themenspektrum des Kinder- und Jugendsports abzubilden und wünschen Ihnen und euch neue Informationen und Anregungen und viel Freude beim Lesen.

Michael Leyendecker (für die Institutionellen Herausgeber*innen)

Nils Neuber (für die Herausgeber*innen der Forschungsbeiträge)

Peter Lautenbach (für die Herausgeber*innen der Fachbeiträge)

